# Genomic Structure, Protein Character, Phylogenic Implication, and Embryonic Expression Pattern of a Zebrafish New Member of Zinc Finger BED-Type Gene Family

**DOI:** 10.3390/genes14010179

**Published:** 2023-01-09

**Authors:** Chih-Wei Zeng, Jin-Chuan Sheu, Huai-Jen Tsai

**Affiliations:** 1 Liver Disease Prevention and Treatment Research Foundation, Taipei 100008, Taiwan; 2 School of Medicine, Fu Jen Catholic University, New Taipei City 242062, Taiwan; 3 Department of Life Science, Fu Jen Catholic University, New Taipei City 242062, Taiwan

**Keywords:** embryonic development, expression pattern, BED-type zinc finger protein, phylogenetic analysis, zebrafish

## Abstract

We reported a new member of the C_2_H_2_-zinc-finger BED-type (ZBED) protein family found in zebrafish (*Danio rerio*). It was previously assigned as an uncharacterized protein LOC569044 encoded by the *Zgc:161969* gene, the transcripts of which were highly expressed in the CNS after the spinal cord injury of zebrafish. As such, this novel gene deserves a more detailed investigation. The 2.79-kb *Zgc:161969* gene contains one intron located on Chromosome 6 at 16,468,776–16,475,879 in the zebrafish genome encoding a 630-aa protein LOC569044. This protein is composed of a DNA-binding BED domain, which is highly conserved among the ZBED protein family, and a catalytic domain consisting of an α-helix structure and an *hAT* dimerization region. Phylogenetic analysis revealed the LOC569044 protein to be clustered into the monophyletic clade of the ZBED protein family of golden fish. Specifically, the LOC569044 protein was classified as closely related to the monophyletic clades of zebrafish ZBED4-like isoforms and ZBED isoform 2. Furthermore, *Zgc:161969* transcripts represented maternal inheritance, expressed in the brain and eyes at early developmental stages and in the telencephalon ventricular zone at late developmental stages. After characterizing the LOC569044 protein encoded by the *Zgc:161969* gene, it was identified as a new member of the zebrafish ZBED protein family, named the ZBEDX protein.

## 1. Introduction

A zinc finger is a small structural motif with one or more coordinated zinc ions (Zn^2+^). The zinc finger protein consists of several structural families based on their different three-dimensional structures, among which the C_2_H_2_-type zinc finger motif is the most common structure among the classic C_2_H_2_-type zinc finger proteins. Some C_2_H_2_-type zinc finger proteins were reported to play an important role in zebrafish development. For example, the C_2_H_2_-type zinc finger protein 16-like protein regulates oligodendrocyte specification, migration, and myelination during zebrafish embryo development [1]. The C_2_H_2_-type zinc finger protein insulinoma-associated 1 promotes zebrafish CaP motor neuron development through Olig2 and Nkx6.1 [2], and the C_2_H_2_-type zinc finger transcription factor (sp7) promotes skeletogenesis [3]. 

The C_2_H_2_-zinc-finger BED-type (ZBED) gene represents another zinc finger protein family type. The ZBED protein family is widely expressed in vertebrate tissues, consisting of numerous genes and sharing a unique and conserved BED domain that binds to the DNA element. This domain was discovered from two chromatin-boundary-element-binding proteins, *Drosophila* BEAF and DREF, using bioinformatics analysis [4]. The BED domain comprises 50–60 amino acid residues with two highly conserved aromatic positions, sharing cysteines and histidine to form a zinc finger. Except for the conserved BED domain, each ZBED gene has its own unique domain. Therefore, each member within the ZBED protein family may play its own distinct biological function, e.g., the ZBED2 protein is acquired during pancreatic ductal adenocarcinoma (PDA) progression to suppress the IFN pathway, resulting in the promotion of PDA tumor proliferation [5]. The ZBED3 protein is involved in carcinogenesis and embryogenesis owing to its interaction with Axin, which is vital for Wnt/β–catenin signal modulation in mammals [6]. The *ZBED4* mRNA is exclusively expressed in glial Müller cells and human retina cone photoreceptors [7]. The ZBED6 protein also binds to the target insulin-like growth factor-2 (IGF2) motif and inhibits IGF2 expression, resulting in the promotion of cell proliferation, growth, and development [8]. Therefore, searching for more novel member(s) within the ZBED protein family will allow us to comprehensively understand the family’s characteristics.

In particular, we took advantage of the zebrafish (*Danio rerio*) model to search for a novel member of the ZBED family that controls neurogenesis during the embryonic stage. After analyzing gene information generated from spinal cord-injured zebrafish embryos by RNA-Seq Transcriptome analysis [9], we found a transcript from an unknown zebrafish gene assigned as the *Zgc:161969* gene that was highly expressed in the CNS. Based on amino acid sequence and evolutionary tree analyses, we identified that the uncharacterized LOC569044 protein encoded by the *Zgc:161969* gene was a zebrafish ZBED protein, which turned out to be a new member of the zinc finger BED-type protein family, named zebrafish ZBEDX. Taking this a step further, we clearly defined the *ZBEDX* gene and its encoded ZBEDX protein in terms of genomic and protein information, phylogenetic implication, and embryonic expression patterns. Interestingly, we revealed that *ZBEDX* mRNA is maternally inherited and expressed at high levels during neuronal development at an early embryonic stage.

## 2. Materials and Methods

### 2.1. Ethics Statement

The animal protocol, which was strictly followed in this study, was reviewed and approved by the Institutional Animal Care and Use Committee (IACUC) of the Fu Jen Catholic University with ethics approval number A11064. 

### 2.2. Zebrafish Husbandry and Microscopy

The zebrafish AB strain was cultured as previously described [10]. The embryos were grown at 28.5 °C in embryo media (EM), staged, and fixed as described by Westerfield (2000) [10]. Pigmentation in the embryos was inhibited by supplementing EM with 0.003% 1-phenyl 2-thiourea (PTU) (MiliporeSigma, Darmstadt, Germany) at 24 h post-fertilization (hpf) [11]. The embryos were observed under a light stereomicroscope (MZ FLIII, Leica, Bensheim, Germany).

### 2.3. Sequence Alignments

All published sequences from different species were obtained from GenBank (https://www.ncbi.nlm.nih.gov/genbank/, accessed on 1 March 2021). The deduced amino acid sequences of the ZBED domain of zebrafish Zgc:161969 (uncharacterized protein LOC569044; NP_001077318.1), ZBED1-like (XP_021327416.1), ZBED4 (XP_002666517.2), ZBED4-like (XP_001340716.5), ZBED4-like isoform 1 (XP_005165631.1), ZBED4-like isoform 2 (XP_005165631.1), ZBED isoform 1 (NP_001373713.1) and ZBED isoform 2 (NP_001373714.1) were obtained from the National Center for Biotechnology Information (NCBI) nucleotide and protein database. First, the sequences in the FASTA mode were mapped in a motif search in GenomeNet (https://www.genome.jp, accessed on 1 March 2021) to identify the protein domains and key residues using the Pfam software 35.0 [12] and InterPro database (https://www.ebi.ac.uk/interpro/, accessed on 1 March 2021). Next, we blasted the BED domain in NCBI and identified an uncharacterized protein (LOC569044; GenBank accession number: NM_001083849.1).

### 2.4. Phylogenetic Tree and Homology Analyses

A phylogenetic tree was constructed using the Phylogeny.fr (www.phylogeny.fr, accessed on 1 March 2021) platform based on information about the zebrafish ZBED family from *Ensembl* (www.ensembl.org, accessed on 1 March 2021) and ZFIN (www.zfin.org, accessed on 1 March 2021). MUSCLE v5, DNA Data Bank of Japan (DDBJ), and ClustalW/X (the version is 2.1) were utilized for multiple alignments: PhyML for tree building and TreeDyn for tree drawing [13,14]. The amino acid sequence encoded by *Zgc:161969* (NP_001077318.1) was deduced, and its phylogenetic tree was determined in two ways, as follows:

(1) Based on the full-length amino acid sequence: the alignment between LOC569044 and other members in the ZBED family of known species, including *Homo sapiens* ZBED4 (AAI67155.1), E3 SUMO-protein ligase ZBED1 (NP_001164606.1) and ZBED1 (AAH15030.1); *Rattus norvegicus* ZBED4 (XP_032775174.1); *Mus musculus* ZBED4 (NP_852077.1); *Xenopus tropicalis* ZBED1 (XP_004911791.1), and ZBED1 isoform X1 (XP_012813067.2); *Carassius auratus* ZBED1-like (XP_026072939.1), ZBED1-like isoform X1 (XP_026117831.1), ZBED1-like isoform X2 (XP_026117841.1), ZBED4-like (XP_026116228.1), ZBED4-like isoform X1 (XP_026069790.1), ZBED4-like isoform X2 (XP_026069791.1), ZBED4-like isoform X3 (XP_026117848.1), and *Triplophysa tibetana* ZBED4 (KAA0713175.1).
(2) Based on the BED domain: the alignment between LOC569044 and other known species, including *H. sapiens* ZBED5 (NP_001137139.1), Transposase-like protein (AAF18454.1), Transcription factor IIIA (NP_002088.2), DNA/RNA-binding protein (AAA75623.1), and *Xenopus* transcription factor IIIA homologue (BAA06988.1); *M. musculus* Transcription factor IIIA isoform CRA a (EDL05819.1) and Transcription factor IIIA isoform CRA b (EDL05820.1); *X. tropicalis* PR domain zinc finger protein 15 isoform X1 (XP_012813515.1); *C. auratus* ZBED1-like (XP_026072939.1), ZBED4-like isoform X1 (XP_026117831.1), Trichohyalin-like (XP_026072553.1), ZBED1-like isoform X1 (XP_026117831.1), Zinc finger protein 319-like isoform X1 (XP_026084591.1) and Putative transposase (AFC96943.1); *T. tibetana* ZBED4 (KAA0712498.1), and Zinc finger protein 1 (KAA0710290.1).

### 2.5. Protein Structure Prediction

The topological elements of secondary and tertiary structures of ZBED proteins were predicted with Robetta (http://robetta.bakerlab.org/, provided by the Baker lab, HHMI’s Janelia Research Campus, accessed on 1 August 2021) [15] using default parameters.

### 2.6. RNA Extraction, cDNA Synthesis, Polymerase Chain Reaction (PCR), and Antisense mRNA Probe Synthesis

The tissues were homogenized, frozen in TRIzol reagent (15596026, Invitrogen), and stored at −80 °C. The total RNA extraction was performed according to Zeng et al. (2022) [16,17]. In summary, 2 μg of RNA were reverse transcribed into cDNA using the Transcriptor First Strand cDNA Synthesis Kit (Roche Molecular Systems, Pleasanton, CA, USA). The synthesized cDNA was stored at −20 °C. All PCR amplifications were carried out in 50-μL reactions using specific primers. We generated the RNA probe by reverse transcribing the DNA fragments already cloned into a pCS2 vector. For ZBEDX, 1800 base pairs were amplified by PCR using the forward primer (5′-TATATCTAGAGGATCCATGGAGAGATCTCGTACAGC-3′) and reverse primer (5′-TATACTCGAGTTATTCCAAAGTGGAGATGATTTTGC-3′). The gene encoding this protein was subsequently cloned.

### 2.7. Whole-Mount In Situ Hybridization (WISH)

WISH was performed as described by Zeng et al. (2016) [18] and Lee et al. (2017) [19] with some modifications. Digoxigenin (DIG)-labeled RNA antisense probe was synthesized from linearized plasmid using the DIG RNA Labeling Kit (SP6/T7) (Roche). After permeabilization, zebrafish embryos at the stages of 1-cell, 2-cell, 24-, 48-, 72-, and 96-hpf were hybridized with the probe overnight and incubated with an anti-DIG antibody (Roche; 1:8000 times dilution). Positive signals were observed under a light stereomicroscope (MZ FLIII, Leica, Bensheim, Germany).

## 3. Results and Discussion

### 3.1. A New Member of the ZBED Protein Family Encoded by Zebrafish Zgc:161969

Gene *Zgc:161969* was screened from the RNA-seq transcriptomic source generated by the spinal cord injury of zebrafish. Since it showed a highly increased expression during spinal cord injury, it was determined to be involved in neuronal regeneration [9] for this study. However, neither the *Zgc:161969* gene nor its encoded protein LOC569044 has been completely characterized: a task we performed in this work. 

**Figure 1:**
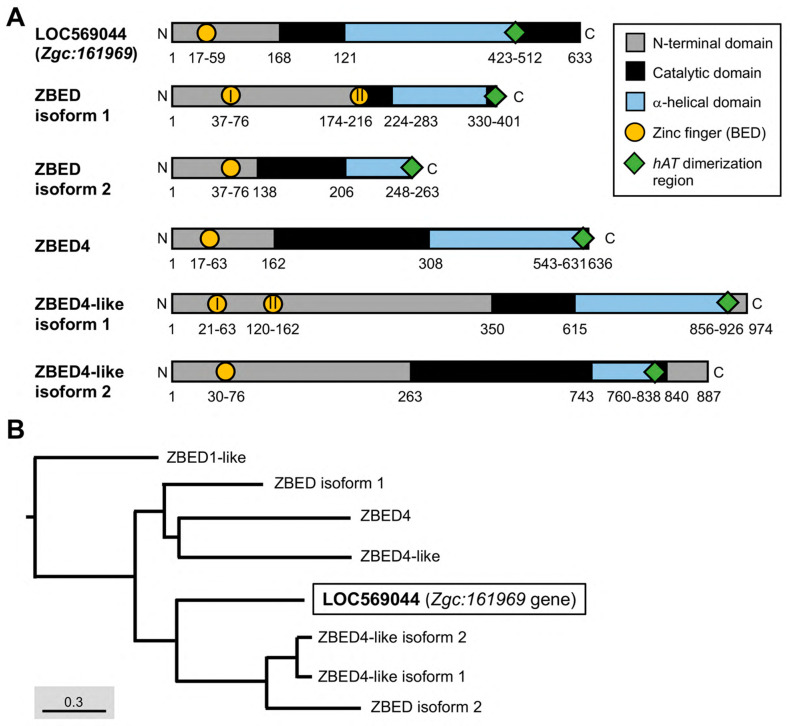
The essential domains of the ZBED protein family in zebrafish and their phylogenetic tree. (**A**) Different domains were labeled by various colors as indicated. Arabic numbers represent the position of amino acid residues located at each domain, while Roman numerals presented in circles indicate the number of zinc finger BED domains existing in ZBED proteins. (**B**) Phylogenetic tree for ZBED proteins in zebrafish (transcript ID in Table 1). Three monophyletic clades indicated by different colors are categorized. The uncharacterized LOC569044 protein encoded by the *Zgc:161969* gene is marked by a black box.

The 2.79-kb *Zgc:161969* gene consists of one intron located on chromosome 6 at 16,468,776–16,475,879 in the zebrafish genome (Ensembl number ENSDARG00000041359.7). The full-length cDNA of *Zgc:161969* contains 1890 nucleotide base pairs (bp) encoding 633 amino acid residues [National Center for Biotechnology Information (NCBI) GenBank accession number: NM_001083849.1; Figure 1A]. Using *Pfam* software and the *InterPro* database to predict putative major domains, we found that the LOC569044 protein encoded by *Zgc:161969* contained a ZBED domain located at amino acid residues from 17 to 59 (Figure 1A).

Upon checking both potential transcripts from NCBI, the result showed that both transcripts XM_005168715 and NM_0010838491 are predicted from *D. rerio zgc:161969* (*zgc:161969*), transcript variant X1, mRNA (717 amino acids), *D. rerio zgc:161969* (*zgc:161969*), and mRNA (630 amino acids), respectively. After using the standard protein BLAST of both proteins predicted from the NCBI database, we found that the percentage of the full-length NM_0010838491 protein alignment with XM_005168715 was a 93% identity. The alignment result showed that the different region between them was only a few amino acids located at the N-terminal site (Supplementary Figure S1; green color). Since both potential transcripts contain an essential ZBED domain (Supplementary Figure S1; yellow color), we suggested that both potential transcripts belonged to the ZBED protein family. However, those different parts of sequences are still undefined, which might provide us with an interesting topic for future study. In this study, we used XM_005168715 and NP_001077318.1 as reference sequences from NCBI. 

To future investigate whether the *Zgc:161969* gene is synteny in conservation across bony fishes or to other vertebrates, we performed the genomic alignments of 65 bony fishes. The results revealed a very low degree of synteny between the zebrafish LOC569044 protein and that of the other bony fish we compared. However, a little similar alignment sequence located at chromosome 6 was found in three species of bony fish, which included *Sinocyclocheilus grahami*, *Astyanax mexicanus,* and *Ictalurus punctatus* (Supplementary Figure S2). Additionally, we could not find any syntenic conservation in humans. Based on these results, we suggested that (1) the *Zgc:161969* has a very low degree of synteny conservation across bony fishes and other vertebrates, and (2) the *Zgc:161969* has its own unique amino acid sequence.

Since the LOC569044 protein encoded by *Zgc:161969* in zebrafish possesses a BED domain which is an essential and evolutionarily conserved DNA-binding domain in the ZBED protein family, we further characterized the biological property of this protein using a comparative protein approach. We first examined whether the LOC569044 protein encoded by the *Zgc:161969* gene co-evolved with other proteins in the zebrafish ZBED protein family. After the NCBI Standard protein BLAST of more than 20 zebrafish ZBED proteins predicted from the NCBI database, we did not align any known ZBED proteins that shared a highly similar protein sequence, suggesting that the LOC569044 protein encoded by *Zgc:161969* has its own unique amino acid sequence, except for containing a BED domain which is a highly conserved domain within the zebrafish ZBED protein family (Figure 1B). When the deduced amino acid sequence of LOC569044 was compared with that of all known zebrafish ZBED proteins, we found that it shared 37, 67, 58, 50, 50, 52, and 54% identity with zebrafish ZBED1-like, ZBED4, ZBED4-like, ZBED4-like isoform 1, ZBED4-like isoform 2, ZBED isoform 1, and ZBED isoform 2, respectively (Table 1). Specifically, the protein sequence of LOC569044 was relatively similar to that of ZBED4 and ZBED4-like isoforms. On the other hand, when the phylogenetic analysis was performed, we found that two major monophyletic clades were categorized within the ZBED protein family of the zebrafish and that the LOC569044 protein was classified as related to the monophyletic clades of ZBED4-like isoforms and ZBED isoform 2 (Figure 1A).

Except for the typical BED domain, we went further to analyze another conserved domain, the *hAT* dimerization region, of the LOC569044 protein and compared it with that of zebrafish ZBED proteins. It has been reported that ZBED proteins may have been domesticated from transposases in one or two independent regions [4]. DNA transposon in the *hAT* superfamily is widespread in animals, including several active and well-studied elements, such as the Ac transposon of *Zea mays*, the hobo transposon of *Drosophila melanogaster*, the Hermes transposon of the housefly *Musca domestica,* and the Tol2 transposon of the Japanese medaka fish (*Oryzias latipes)* [20,21]. When we compared the LOC569044 protein with other ZBED proteins in zebrafish using Pfam software 35.0, we found that the LOC569044 protein contained a *hAT* dimerization domain located at amino acid residues from 423 to 512 (Figure 1B). The *hAT* transposons and related domesticated sequences constitute a large superfamily that was recently characterized [22]. Similar to many members of the zebrafish ZBED family proteins, the LOC569044 protein was found to belong to the *hAT* transposon superfamily. 

Based on protein sequence identity, conserved BED and *hAT* dimerization domains, and phylogenetic analysis, we concluded that the LOC569044 protein encoded by *Zgc:161969* is a new member of the ZBED protein family in zebrafish, designated as the ZBEDX protein. 

### 3.2. Prediction of ZBEDX Protein Structure

**Figure 2:**
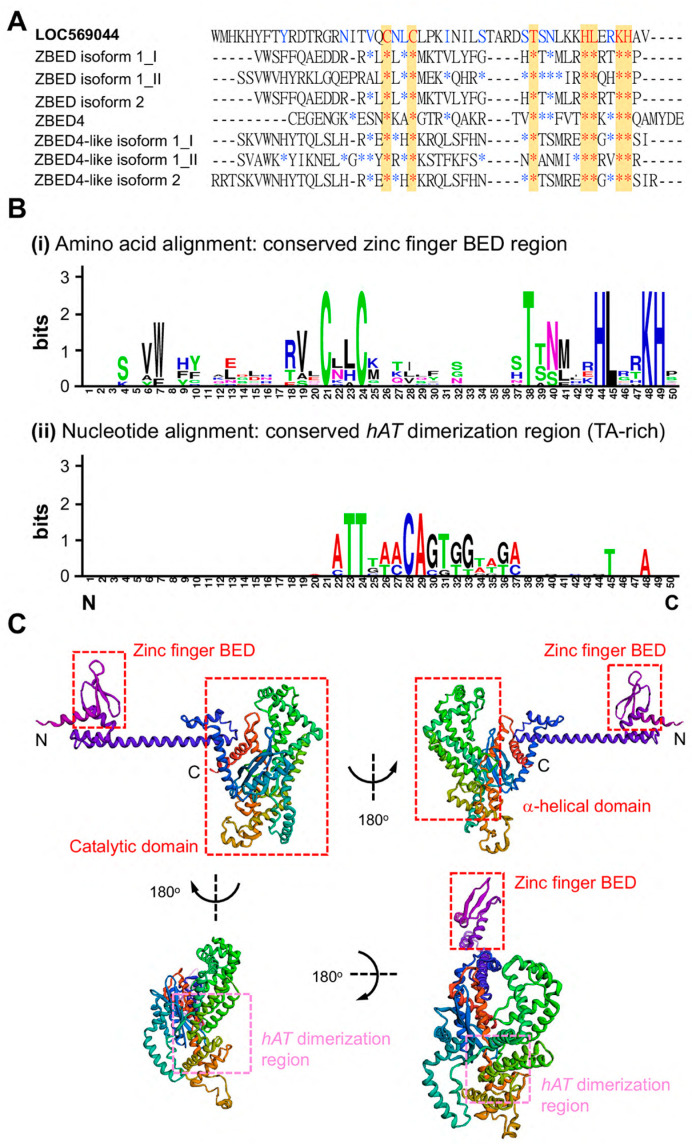
Comparison of the amino acid sequences of ZBED domain between LOC569044 protein and other zebrafish ZBED proteins. (**A**) The alignment of amino acids of BED domains among zebrafish ZBED proteins. The DNA-binding BED domain was characterized by the signature C^x2^C^xn^H^x3-5^H (xn indicates a variable spacer; yellow color). Blue color indicates that at least one amino acid residue among zebrafish ZBED proteins was identical to that of LOC569044 protein, while the red color indicates amino acid residue in all zebrafish ZBED proteins entirely identical to that of the LOC569044 protein. *: indicates that the amino acid residue is identical among all species examined. (**B**) Using the WebLogos (version 2.8.2) to analyze the consensus residues of amino acid and nucleic acid of conserved zinc finger BED domain (**i**) and *hAT* dimerization region (**ii**), respectively. The larger size of alphabetic letters represent a higher degree of consensus. (**C**) A predicted protein structure of LOC569044 protein. Four views of the predicated protein structures were demonstrated by a 180-degree rotation. Positions of N- and C-termini are labeled. Positions of the three domains are labeled by red boxes, and a *hAT* dimerization region is labeled by pink box.

ZBED proteins have recently been characterized by signature C^x2^C^xn^H^x3−5^[H/C] (xn is a variable spacer). The amino acid sequence of the BED domain of the ZBED protein was compared with that of seven ZBED proteins in zebrafish, such as ZBED isoform 1_I (first ZBED domain), ZBED isoform 1_II (second ZBED domain), ZBED isoform 2, ZBED 4, and ZBED4-like isoform 1_I (Figure 2A). Based on the amino acid sequence alignment of the zinc-finger regions of these seven ZBED proteins in zebrafish, the conserved region C^x2^C^xn^T^xn^H^x4^L^x2^KH was found to be shared (Figure 2A, red star and yellow). We noticed that the zinc-finger region of this novel ZBEDX protein also contained the conserved C^2^C^19^T^5^HL^2^KH region.

We also employed WebLogos (version 2.8.2, https://weblogo.berkeley.edu/, accessed on 1 August 2021) to compare the consensus amino acid residue of the ZBEDX protein and seven other known ZBED proteins in zebrafish. The results demonstrated that similar to the seven ZBED proteins in zebrafish, ZBEDX contains a conserved zinc-finger BED domain-containing seven consensus amino acid residues CCTHLTH (Figure 2(Bi)). Additionally, when we compared the nucleic acid sequence of the *hAT* dimerization region of the ZBEDX protein with those of seven ZBED proteins in zebrafish, four consensus nucleic acids TTAA (TA-rich) were found (Figure 2(Bii)). A TA-rich sequence is required for the integration of each transposon. Thus, a greater number of TA-rich consensus sequences should enhance the chance that transposon could have in finding a site to integrate [23]. The recent characterization of ZBED proteins shows the presence of two highly conserved aromatic amino acids: tryptophan and phenylalanine [24]. However, ZBEDX contains 5% phenylalanine and 1% tryptophan, suggesting that the ZBEDX protein contains one zinc-finger region of the BED domain, a TA-rich *hAT* dimerization region, and a highly conserved aromatic amino acid, phenylalanine.

In general, the main function of the zinc-finger region of the BED domain is to bind DNA elements. However, its other functions include the binding of RNA, proteins, and small molecules [25]. In fact, structural changes in the BED domain might alter the binding specificity of a particular protein [26]. Therefore, the secondary structure of the ZBED protein might help us understand how ZBED interacts with DNA, RNA, or proteins. We investigated protein structure prediction using *Robetta* (see Materials and Methods), and the ZBEDX protein-predicted model was found to be a helix-rich representative structural model separated into two major regions (Figure 2C). One was an N-terminal BED domain consisting of a helix–turn–helix motif near the N-terminus formed by amino acid residues from 1 to 168 and a zinc-finger region. The zinc-finger region could be further classified into several structural families, and each one has a unique three-dimensional architecture [27]. Therefore, we speculated that the helix–turn–helix motif of ZBEDX may be a binding region for DNA, RNA, or proteins. Another region was the catalytic domain consisting of several helix–turn–helix motifs containing β-sandwich structures near the C-terminus formed by amino acid residues from 169 to 633. This domain included over ten helix–turn–helix motifs and was composed of α-helical domains and a *hAT* dimerization region which may require the activation, repression, or epigenetic control of DNA [22]. This line of evidence indicated that the ZBEDX protein contained a BED domain at the N-terminal domain, as well as α-helical domains and a *hAT* dimerization region at the catalytic domain, suggesting that the predicted secondary structure of ZBEDX was consistent with the secondary structures of other proteins within the ZBED family.

### 3.3. Phylogenetic Analysis of Zebrafish ZBEDX Protein

**Figure 3:**
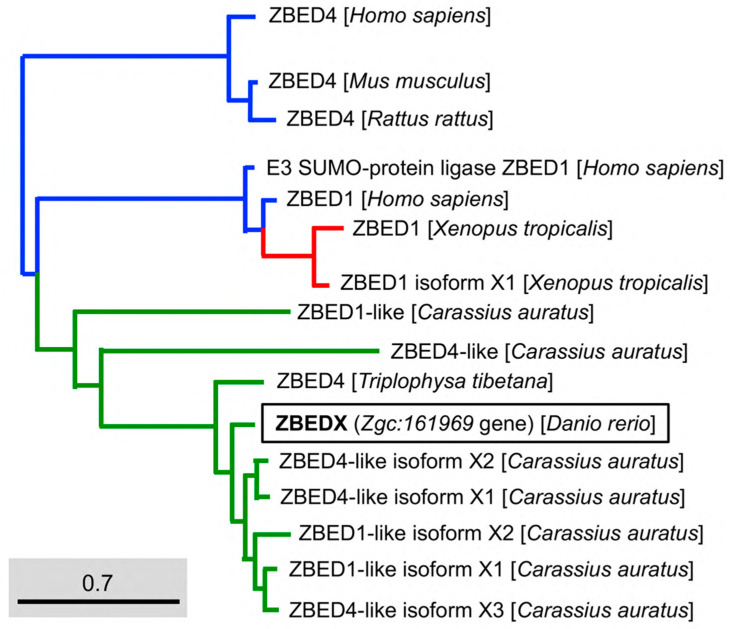
Phylogenetic analysis of the LOC569044 protein and the ZBED proteins found in mammals, amphibians, and bony fish. The full amino acid sequences of ZBED proteins from the known species are human (*H. sapiens*), mouse (*M. musculus*), zebrafish (*D. rerio*), frog (*X. tropicalis*), goldfish (*C. auratus*), and stone loach (*T. tibetana*), which were used to characterize the LOC569044 protein. ZBED proteins from known species were categorized into three main monophyletic clades labeled in different colors (transcript ID in Table 2). The LOC569044 protein marked by a black box was categorized in the same monophyletic group, along with the ZBED of bony fish (in green). The scale bar represents the number of substitutions per site. The blue and red colors indicate the same monophyletic group in the mammals and amphibians in, respectively.

To examine the evolutionary relationship between teleost and other species of the ZBED family, we constructed a phylogenetic tree based on the full-length deduced entire amino acid sequence of the zebrafish ZBEDX protein. The results demonstrated that the zebrafish ZBEDX found in this study were clustered into the *Osteichthyes* (bony fish) monophyletic group (Figure 3). When the deduced amino acid sequence of zebrafish ZBEDX was compared with sequences of other species in the ZBED family, we found that the zebrafish ZBEDX shared (1) 22, 75, 75, 20, 75, 82, and 81% identity with bony fish *C. auratus* ZBED1-like, ZBED1-like isoform X1, ZBED1-like isoform X2, ZBED4-like, ZBED4-like isoform X1, ZBED4-like isoform X2, and ZBED4-like isoform X3, respectively. In addition, it shared (2) 21 and 20% identity with amphibian *X. tropicalis* ZBED1, and ZBED1 isoform X1, respectively; (3) 20, 24, and 21% identity with human Homo sapiens ZBED4, E3 SUMO-protein ligase ZBED1 and ZBED1, respectively; and (4) 21% identity with *M. musculus* and *Rattus rattus* (Table 2). Based on these results, we concluded that zebrafish ZBEDX is evolutionarily close to the ZBED family of bony fish. 

**Figure 4:**
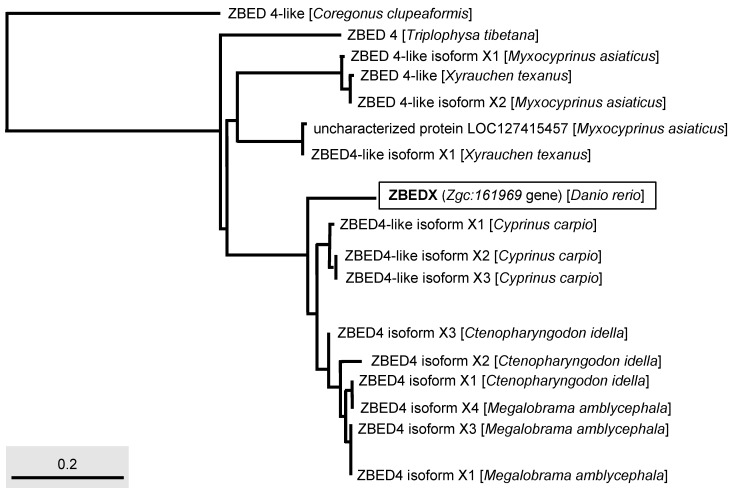
Phylogenetic analysis of the LOC569044 protein and ZBED protein found in multiple bony fishes. The full-length amino acid sequences of ZBED proteins from different species of bony fishes, such as *C. clupeaformis*, *T. tibetana*, *M. asiaticus*, *X. texanus*, *C. carpio*, *C. idella,* and *M. amblycephala* used to characterize the LOC569044 protein. The LOC569044 protein is marked by a rectangular box. The scale bar represents the number of substitutions per site.

Next, we used phylogenetic analysis to compare the ZBEDX and ZBED proteins found in multiple bony fishes. The result showed the ZBEDX clustered into ZBED4 monophyletic group (Figure 4). However, ZBEDX was a more distantly related group of organisms (outgroup), without any ZBED protein in the same clade, suggesting that zebrafish ZBEDX has its own unique amino acid sequence not only presented in zebrafish per se but also presented in other bony fish (Figure 4). After the Standard protein BLAST of ZBED proteins of multiple bony fishes predicted from the NCBI database, we found that the percentage of the full-length ZBEDX protein alignment with multiple bony fishes were 84, 83, and 83% identity with the *Ctenopharyngodon idella* ZBED4 isoform X2, *Cyprinus carpio* ZBED4-like isoform X2, and ZBED4-like isoform X3, respectively (Table 3). Taken together, we conclude that zebrafish ZBEDX is (1) evolutionarily close to the ZBED family of bony fishes and (2) clustered into the monophyletic clade and outgroup of the ZBED protein family of multiple bony fishes.

The number of BED domains varies among ZBED family proteins in zebrafish. For example, ZBED isoform 2, ZBED4, and ZBED4-like isoform 2 contain a single BED domain, while ZBED isoform 1 and ZBED4-like isoform 1 contain two BED domains (Figure 1A). The new member ZBEDX protein discovered in this study contains a single BED domain similar to that of ZBED isoform 2, ZBED4, and ZBED4-like isoform 2. However, the number of BED domains among ZBED family proteins in humans is relatively wide-ranging. For example, human ZBED1 contains a single BED domain, ZBED6 has two BED domains, and ZBED4 contains four BED domains, while the human ZBED6 C-terminal-like protein (C7ORF29) is completely absent of BED domain, possibly from the loss of its original BED domain or the failure to insert ancestrally active transposons during evolution [28]. Thus, investigating the evolutionary mechanisms leading to the occurrence of variable numbers of BED domains among the ZBED family proteins would be an interesting question for further study. 

**Figure 5:**
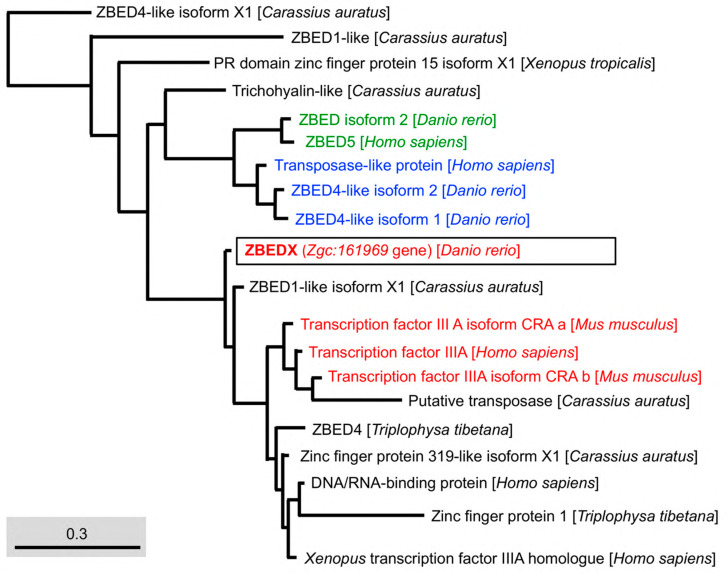
Phylogenetic analysis of the LOC569044 protein and the ZBED domains found in mammals, amphibians, and bony fish. The full amino acid sequences of ZBED domains in ZBED proteins from the known species are human (*H. sapiens*), mouse (*M. musculus*), zebrafish (*D. rerio*), frog (*X. tropicalis*), goldfish (*C. auratus*), and stone loach (*T. tibetana*), which were used to characterize the LOC569044 protein. The LOC569044 protein is marked by a black box. The proteins labeled in the same color are recognized as a group of proteins that are close in evolution and function of BED domains. Scale bar represents the number of substitutions per site.

Regarding the context of the BED domain, we employed phylogenetic trees to analyze the amino acid sequences of the BED domain of mammals, amphibians, and bony fish. The results demonstrated that the BED domain of ZBEDX was categorized into a specific monophyletic clade in which the transcription factor IIIA family of mammals, such as the *M. musculus* Transcription factor IIIA (TFIIIA) isoform CRA and *H. sapiens* TFIIIA, was included (Figure 5). The human TFIIIA is a single protein that contains zinc and possesses a repetitive C2H2 zinc finger domain to regulate the transcription of the 5S ribosomal RNA gene [29]. On the other hand, we noticed that the BED domain of zebrafish ZBEDX shared the same monophyletic clade with that of zebrafish ZBED4-like isoforms and ZBED isoform 2 (Figure 1). However, the BED domains of ZBED4-like isoforms and ZBED isoform 2 were also grouped into the same monophyletic clade as that of the human Transposase-like protein (Figure 5). Although the teleost and other vertebrates contain a conserved BED domain among ZBED proteins categorized into the same monophyletic clade, the above results suggest that the total amino acid sequence of the BED domain is not necessarily identical or even similar, resulting in different biological functions. 

### 3.4. Temporospatial Expression of ZBEDX mRNA in Zebrafish Embryos

**Figure 6:**
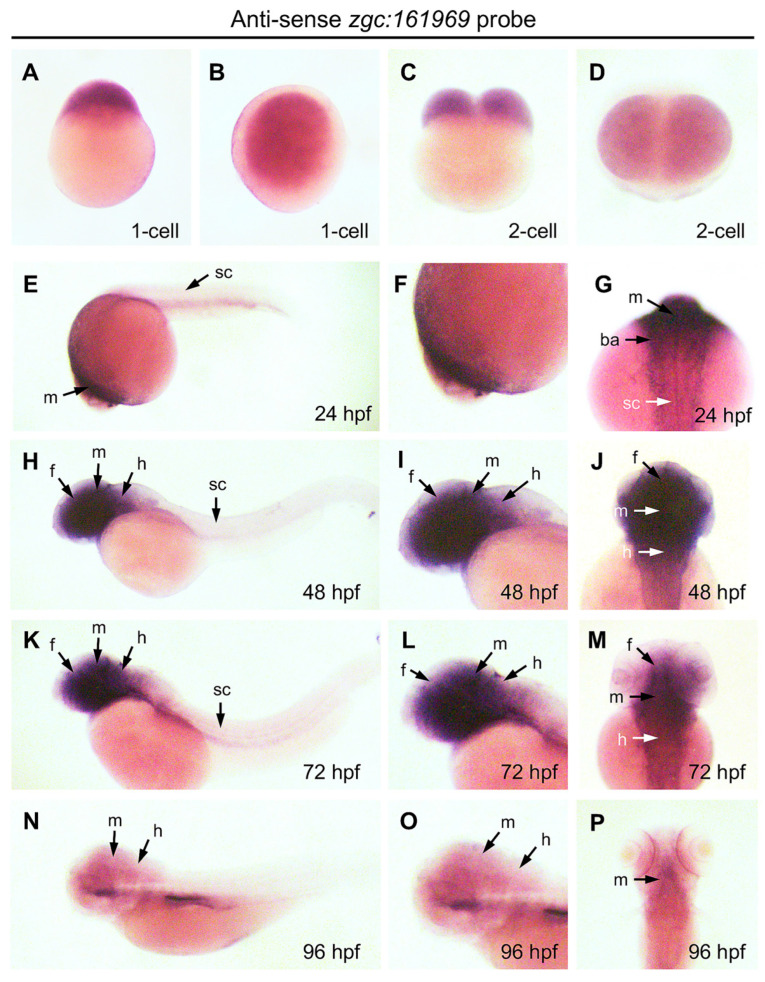
Expression pattern of *ZBEDX* transcripts during the early developmental stages of zebrafish embryos. Embryos at different stages, as indicated, were collected and hybridized with antisense probe against ZBEDX mRNA using whole-mount in situ hybridization. Panels (**A**,**C**,**E**,**H**,**K**,**N**) are lateral views, while panels (**B**,**D**,**F**,**I**,**J**,**L**,**M**,**O**,**P**) are dorsal views. Panels (**E**,**H**,**K**,**N**) are lateral views, and the anterior of embryos was placed at the left, while panels (**G**,**J**,**M**,**P**) are dorsal views, and the anterior of embryos is on the top. At 12 hpf, ZBEDX transcripts were expressed in whole embryo. ZBEDX transcripts were initially apparent at the 1- (**A**,**B**) and 2-cell (**C**,**D**) stages. At 24–72 hpf, ZBEDX transcripts were expressed in forebrain (f), midbrain (m), hindbrain (h), retina (r), spinal cord (sc), and branchial arches (ba) (**E**–**M**). At 96-hpf, ZBEDX transcripts were expressed in midbrain (m) and hindbrain (h) (**N**–**P**).

To examine the temporospatial expression of zebrafish *ZBEDX* transcripts, WISH was performed on zebrafish embryos collected from 24, 48, 72, and 96 hpf. The data demonstrated that zebrafish *ZBEDX* transcripts were detectable as early as the one- and two-cell stages (Figure 6A–D), suggesting that *ZBEDX* mRNAs were transmitted into oocytes through maternal inheritance. Later on, from 24 to 72 hpf, *ZBEDX* transcripts were specifically distributed in the forebrain, midbrain, hindbrain, retinae, and branchial arches (Figure 6E–M). Since *ZBEDX* transcripts were detected in the central nervous system and eyes during embryonic development, it is highly likely that the ZBEDX protein might be involved in developing these tissues during embryogenesis in zebrafish. Moreover, *ZBEDX* transcripts were detected explicitly in the ventricular zone (VZ) of the brain at 48 hpf (Figure 6N–P). At 72 hpf, *ZBEDX* transcripts were mainly displayed in the telencephalon VZ, where cells proliferated actively before eventually migrating to the mantle zone [30]. Some embryonic neuronal stem/progenitor cells (NSPCs) in VZ may also possess similar proliferation.

## 4. Conclusions

This study is the first report to define a previously uncharacterized LOC569044 protein encoded by the uncharacterized zebrafish gene *Zge:161969*. Based on the deduced amino acid sequence alignment, protein structure, and phylogenetic analyses, we confirmed that the LOC569044 protein is (1) composed of a DNA-binding BED domain and a catalytic domain; (2) clustered into the monophyletic clade of the ZBED protein family of fish; and (3) classified as closely related to the monophyletic clades of zebrafish ZBED4-like isoforms and ZBED isoform 2. This line of evidence suggests that the zebrafish LOC569044 protein encoded by the *Zge:161969* gene is a new member of the zebrafish zinc finger BED-type protein family, designated as the zebrafish ZBEDX protein. Meanwhile, we also studied the expression patterns of *ZBEDX* transcripts in the zebrafish embryos at various developmental stages and found that they are mainly distributed in the brain, spinal cord, and eyes. Specifically, *ZBEDX* transcripts are maternally inherited.

## Figures and Tables

**Table 1:** The identity (in percentage) of deduced amino acid residues of novel LOC569044 protein compared with those of seven known ZBED proteins found in zebrafish.

Species	Name	Transcript ID	Length (aa)	Total Identity (%) ^a^	BED Domain Identity (%) ^b^
*D. rerio*	LOC569044 (*Zgc:161969* gene)	BC134839.1	630	100	100
	ZBED1-like	XP_021327416.1	661	19	37
	ZBED4	XP_002666517.2	636	25	67
	ZBED4-like	XP_001340716.5	650	20	58
	ZBED4-like isoform 1	XP_005165631.1	976	26	50
	ZBED4-like isoform 2	XP_005165631.1	887	26	50
	ZBED isoform 1	NP_001373713.1	401	34	52
	ZBED isoform 2	NP_001373714.1	263	26	54

Note: ^a^ The query cover described how many (in %) amino acid (aa) residues in the full-length of novel LOC569044 protein are identical with those of the known zinc finger BED domain-containing protein members in zebrafish; ^b^ The identity (in %) of aa residues contained in the BED domain among zebrafish ZBED proteins.

**Table 2:** The identity (in percentage) of deduced amino acid residues of the ZBEDX protein compared with those of known ZBED proteins found in higher vertebrates.

Species	Name	Transcript ID	Length (aa)	Identity (%) ^a^
*D. rerio*	ZBEDX (*Zgc:161969* gene)	NP_001077318.1	630	100
*C. auratus*	ZBED1-like	XP_026072939.1	628	22
	ZBED1-like isoform X1	XP_026117831.1	729	75
	ZBED1-like isoform X2	XP_026117841.1	667	75
	ZBED4-like	XP_026116228.1	631	20
	ZBED4-like isoform X1	XP_026069790.1	722	75
	ZBED4-like isoform X2	XP_026069791.1	635	82
	ZBED4-like isoform X3	XP_026117848.1	642	81
*H. sapiens*	ZBED4	AAI67155.1	1171	20
	E3 SUMO-protein ligase ZBED1	NP_001164606.1	694	24
	ZBED1	AAH15030.1	694	21
*M. musculus*	ZBED4	NP_852077.1	1168	21
*R. rattus*	ZBED4	XP_032775174.1	1170	21
*X. tropicalis*	ZBED1	XP_004911791.1	639	21
	ZBED1 isoform X1	XP_012813067.2	693	20

Note: ^a^ The query cover describes how many (in %) amino acid (aa) residues in the full-length of novel ZBEDX protein are identical with those of known BED domain-containing ZBED proteins found in higher vertebrates.

**Table 3:** The identity (in percentage) of deduced amino acid residues of the ZBEDX protein compared with those of known ZBED proteins found in bony fishes.

Species	Name	Transcript ID	Length (aa)	Identity (%) ^a^
*D. rerio*	ZBEDX (*Zgc:161969* gene)	NP_001077318.1	630	100
*Coregonus clupeaformis*	ZBED4-like	XP_041752989.2	623	51
*T. tibetana*	ZBED4	KAA0713175.1	622	68
*Myxocyprinus asiaticus*	ZBED 4-like isoform X1	XP_051540434.1	637	69
	ZBED 4-like isoform X2	XP_051540435.1	636	69
	uncharacterized protein LOC127415457	XP_051510176.1	627	67
*Xyrauchen texanus*	ZBED 4-like	XP_051973224.1	636	69
	ZBED4-like isoform X1	XP_051950732.1	627	67
*C. idella*	ZBED4 isoform X1	XP_051752038.1	749	78
	ZBED4 isoform X2	XP_051752039.1	662	84
	ZBED4 isoform X3	XP_051752040.1	654	78
*Megalobrama amblycephala*	ZBED4 isoform X1	XP_048056422.1	730	78
	ZBED4 isoform X3	XP_048056424.1	724	78
	ZBED4 isoform X4	XP_048056425.1	716	78
*C. carpio*	ZBED4-like isoform X1	XP_042613966.1	722	77
	ZBED4-like isoform X2	XP_018955884.1	635	83
	ZBED4-like isoform X3	XP_018927958.2	637	83

Note: ^a^ The query cover describes how many (in %) amino acid (aa) residues in the full-length of novel ZBEDX protein were identical with those of known BED domain-containing ZBED proteins found in higher vertebrates. The gray column shows the top three high identity percentages of aa residues in the full-length novel ZBEDX protein compared to multiple bony fishes.

## Data Availability

The data supporting the findings of this study are available from the corresponding authors upon request.

## Supplementary Materials

The following supporting information can be downloaded at: https://www.mdpi.com/article/10.3390/genes14010179/s1, Figure S1. The deduced amino acid sequences of two potential transcripts and one uncharacterized protein LOC569044; Figure S2. Comparison of the gene maps encoding zebrafish LOC569044 protein and those of multiple bony fishes.

## References

[1] Sidik H., Talbot W.S. (2015). A Zinc Finger Protein That Regulates Oligodendrocyte Specification, Migration, and Myelination in Zebrafish. Development.

[2] Gong J., Wang X., Zhu C., Dong X., Zhang Q., Wang X., Duan X., Qian F., Shi Y., Gao Y. (2017). Insm1a Regulates Motor Neuron Development in Zebrafish. Front. Mol. Neurosci..

[3] DeLaurier A., Eames B.F., Blanco-Sánchez B., Peng G., He X., Swartz M.E., Ullmann B., Westerfield M., Kimmel C.B. (2010). Zebrafish Sp7:EGFP: A Transgenic for Studying Otic Vesicle Formation, Skeletogenesis, and Bone Regeneration. Genesis.

[4] Aravind L. (2000). The BED Finger, a Novel DNA-Binding Domain in Chromatin-Boundary-Element-Binding Proteins and Transposases. Trends Biochem. Sci..

[5] Somerville T.D.D., Xu Y., Wu X.S., Maia-Silva D., Hur S.K., de Almeida L.M.N., Preall J.B., Koo P.K., Vakoc C.R. (2020). ZBED2 Is an Antagonist of Interferon Regulatory Factor 1 and Modifies Cell Identity in Pancreatic Cancer. Proc. Natl. Acad. Sci. USA.

[6] Chen M., Philipp M., Wang J., Premont R.T., Garrison T.R., Caron M.G., Lefkowitz R.J., Chen W. (2009). G Protein-Coupled Receptor Kinases Phosphorylate LRP6 in the Wnt Pathway. J. Biol. Chem..

[7] Saghizadeh M., Akhmedov N.B., Yamashita C.K., Gribanova Y., Theendakara V., Mendoza E., Nelson S.F., Ljubimov A.V., Farber D.B. (2009). ZBED4, a BED-Type Zinc-Finger Protein in the Cones of the Human Retina. Investig. Opthalmology Vis. Sci..

[8] Markljung E., Jiang L., Jaffe J.D., Mikkelsen T.S., Wallerman O., Larhammar M., Zhang X., Wang L., Saenz-Vash V., Gnirke A. (2009). ZBED6, a Novel Transcription Factor Derived from a Domesticated DNA Transposon Regulates IGF2 Expression and Muscle Growth. PLoS Biol..

[9] Zeng C.-W., Kamei Y., Shigenobu S., Sheu J.-C., Tsai H.-J. (2021). Injury-Induced Cavl-Expressing Cells at Lesion Rostral Side Play Major Roles in Spinal Cord Regeneration. Open Biol..

[10] Westerfield M. (2007). The Zebrafish Book: A Guide for the Laboratory Use of Zebrafish (Danio Rerio).

[11] Zeng C.-W., Sheu J.-C., Tsai H.-J. (2020). The Neuronal Regeneration of Adult Zebrafish after Spinal Cord Injury Is Enhanced by Transplanting Optimized Number of Neural Progenitor Cells. Cell Transplant..

[12] Bateman A. (2002). The Pfam Protein Families Database. Nucleic Acids Res..

[13] Dereeper A., Guignon V., Blanc G., Audic S., Buffet S., Chevenet F., Dufayard J.-F., Guindon S., Lefort V., Lescot M. (2008). Phylogeny.fr: Robust Phylogenetic Analysis for the Non-Specialist. Nucleic Acids Res..

[14] Zeng C.-W., Sheu J.-C., Tsai H.-J. (2020). A New Member of the Forkhead Box Protein Family in Zebrafish: Domain Composition, Phylogenetic Implication and Embryonic Expression Pattern. Gene Expr. Patterns.

[15] Kim D.E., Chivian D., Baker D. (2004). Protein Structure Prediction and Analysis Using the Robetta Server. Nucleic Acids Res..

[16] Zeng C.-W., Sheu J.-C., Tsai H.-J. (2022). Hypoxia-Responsive Subtype Cells Differentiate into Neurons in the Brain of Zebrafish Embryos Exposed to Hypoxic Stress. Cell Transplant..

[17] Lin C.-Y., He J.-Y., Zeng C.-W., Loo M.-R., Chang W.-Y., Zhang P.-H., Tsai H.-J. (2017). MicroRNA-206Modulates an Rtn4a/Cxcr4a/Thbs3a Axis in Newly Forming Somites to Maintain and Stabilize the Somite Boundary Formation of Zebrafish Embryos. Open Biol..

[18] Zeng C.-W., Kamei Y., Wang C.-T., Tsai H.-J. (2016). Subtypes of Hypoxia-Responsive Cells Differentiate into Neurons in Spinal Cord of Zebrafish Embryos after Hypoxic Stress. Biol. Cell.

[19] Lee H.-C., Fu C.-Y., Zeng C.-W., Tsai H.-J. (2017). Embryonic Expression Patterns of Eukaryotic EndoU Ribonuclease Family Gene EndouC in Zebrafish. Gene Expr. Patterns.

[20] O’brochta D.A., Atkinson P.W. (1996). Transposable Elements and Gene Transformation in Non-Drosophilid Insects. Insect Biochem. Mol. Biol..

[21] Kawakami K., Shima A. (1999). Identification of the Tol2 Transposase of the Medaka Fish Oryzias Latipes That Catalyzes Excision of a Nonautonomous Tol2 Element in Zebrafish Danio Rerio. Gene.

[22] Arensburger P., Hice R.H., Zhou L., Smith R.C., Tom A.C., Wright J.A., Knapp J., O’Brochta D.A., Craig N.L., Atkinson P.W. (2011). Phylogenetic and Functional Characterization of ThehATTransposon Superfamily. Genetics.

[23] Woodard L.E., Li X., Malani N., Kaja A., Hice R.H., Atkinson P.W., Bushman F.D., Craig N.L., Wilson M.H. (2012). Comparative Analysis of the Recently Discovered HAT Transposon TcBuster in Human Cells. PLoS ONE.

[24] Mahajan M.A., Murray A., Samuels H.H. (2002). NRC-Interacting Factor 1 Is a Novel Cotransducer That Interacts with and Regulates the Activity of the Nuclear Hormone Receptor Coactivator NRC. Mol. Cell. Biol..

[25] Klug A. (2010). The Discovery of Zinc Fingers and Their Development for Practical Applications in Gene Regulation and Genome Manipulation. Q. Rev. Biophys..

[26] Laity J.H., Lee B.M., Wright P.E. (2001). Zinc Finger Proteins: New Insights into Structural and Functional Diversity. Curr. Opin. Struct. Biol..

[27] Berg J.M. (1990). Zinc Fingers and Other Metal-Binding Domains. Elements for Interactions between Macromolecules. J. Biol. Chem..

[28] Hayward A., Ghazal A., Andersson G., Andersson L., Jern P. (2013). ZBED Evolution: Repeated Utilization of DNA Transposons as Regulators of Diverse Host Functions. PLoS ONE.

[29] Stünkel W., Kober I., Kauer M., Taimor G., Seifart K.H. (1995). Human TFIIIA Alone Is Sufficient to Prevent Nucleosomal Repression of a Homologous 5S Gene. Nucleic Acids Res..

[30] Grandel H., Kaslin J., Ganz J., Wenzel I., Brand M. (2006). Neural Stem Cells and Neurogenesis in the Adult Zebrafish Brain: Origin, Proliferation Dynamics, Migration and Cell Fate. Dev. Biol..

