# Supplementary material for: Genomic Structure, Protein Character, Phylogenic Implication, and Embryonic Expression Pattern of a Zebrafish New Member of Zinc Finger BED-Type Gene Family
Source: Genes (Basel). 2023 Jan 9;14(1):179. doi: 10.3390/genes14010179 (PMC9859435; doi:10.3390/genes14010179)
Supplement: Supplementary file 1 [file genes-14-00179-s001.zip › genes-2048601-supplementary.pdf]

Supplementary Figures

PREDICTED: Danio rerio zgc:161969 (zgc:161969), transcript variant X1, protein coding

NCBI Reference Sequence: XM_005168715.4

```
MERSRTAFDHWMHKHYFTYRDTRGRNITVQCNLCLPKINILSTARDSTSNLKKHLECHSPHLMRKHQHIEMSEP
EPLFLPPKRLKSAVWEYFGYYKDPGGTIIADGRPVCRMCRKKVSARSGNTSNMQHHLRDHHPMEFAKLNRKHAV
PCRKRLREAAGIDGSSSFATNILENQTEESSEAKDDKNDSTIYYQTSQSRLNALVFNFIVEDVQPISILEQPGF
RKLIEALSRGKRVMSPNAFVTRLEMAFDKMKTELKAKLSKVQTLCTTADIWSVQDRSYFGMTCHWLEDNLERRS
AALACTRIPSNYTCETIIAKIQETHSSFNIESKVQATVTDNGNNFVKAFKEFSSDDEQEVEQSQFEDLGSILCD
GDIGGDFFLSYFLPPHQRCAGQTLTTIASKDLAEAVTKGPAGKVRSSAAGKCAKIWHKAESSMETAVAMESIAN
MKFTLPCLNQWSSEYYAINKLMTLNDSQLNELTELLGVPHFTPDETSYLTEYTDVFKPVAFALDLLQGEEKCFL
GIVIPTLLTLKRKLEEKAANTRLFSKAIEETVKAIDTRFKQVFESSDARLATVTTPQFRLWWLPEEEREVLRAQ
LITEVLQVDQGTEEPETNGGAVHEDEFFSYGPGSPGYKCGKREAAEEVWLYLQGTNKDLKCLNEFPGVKKVFIK
FNTTLPSSAPVQQLFNSGGNNVNPKGNPLTDEQFEHVLLLRYNSKIISALE
```

Danio rerio zgc:161969 (zgc:161969), protein coding [in this study]

NCBI Reference Sequence: NM_001083849.1

```
MERSRTAFDHWMHKHYFTYRDTRGRNITVQCNLCLPKINILSTARDSTSNLKKHLERKHAVPCRKRLREAAGID
GSSSFATNILENQTEESSEAKDDKNDSTIYYQTSQSRLNALVFNFIVEDVQPISILEQPGFRKLIEALSRGKRV
MSPNAFVTRLEMAFDKMKTELKAKLSKVQTLCTTADIWSVQDRSYFGMTCHWLEDNLERRSAALACTRIPSNYT
CETIIAKIQETHSSFNIESKVQATVTDNGNNFVKAFKEFSSDDEQEVEQSQFEDLGSILCDGDIGGDFFLSYFL
PPHQRCAGQTLTTIASKDLAEAVTKGPAGKVRSSAAGKCAKIWHKAESSMETAVAMESIANMKFTLPCLNQWSS
EYYAINKLMTLNDSQLNELTELLGVPHFTPDETSYLTEYTDVFKPVAFALDLLQGEEKCFLGIVIPTLLTLKRK
LEEKAANTRLFSKAIEETVKAIDTRFKQVFESSDARLATVTTPQFRLWWLPEEEREVLRAQLITEVLQVDQGTE
EPETNGGAVHEDEFFSYGPGSPGYKCGKREAAEEVWLYLQGTKKDLKCLNEFPGVKKVFIKFNTTLPSSAPVQQ
LFNSGGNNVNPKGNPLTDEQFEHVLLLRYNSKIISTLE
```

Danio rerio uncharacterized protein LOC569044 [in this study; the sequence same as NM_001083849.1 protein coding]

NCBI Reference Sequence: NP_001077318.1

```
MERSRTAFDHWMHKHYFTYRDTRGRNITVQCNLCLPKINILSTARDSTSNLKKHLERKHAVPCRKRLREAAGID
GSSSFATNILENQTEESSEAKDDKNDSTIYYQTSQSRLNALVFNFIVEDVQPISILEQPGFRKLIEALSRGKRV
MSPNAFVTRLEMAFDKMKTELKAKLSKVQTLCTTADIWSVQDRSYFGMTCHWLEDNLERRSAALACTRIPSNYT
CETIIAKIQETHSSFNIESKVQATVTDNGNNFVKAFKEFSSDDEQEVEQSQFEDLGSILCDGDIGGDFFLSYFL
PPHQRCAGQTLTTIASKDLAEAVTKGPAGKVRSSAAGKCAKIWHKAESSMETAVAMESIANMKFTLPCLNQWSS
EYYAINKLMTLNDSQLNELTELLGVPHFTPDETSYLTEYTDVFKPVAFALDLLQGEEKCFLGIVIPTLLTLKRK
LEEKAANTRLFSKAIEETVKAIDTRFKQVFESSDARLATVTTPQFRLWWLPEEEREVLRAQLITEVLQVDQGTE
EPETNGGAVHEDEFFSYGPGSPGYKCGKREAAEEVWLYLQGTKKDLKCLNEFPGVKKVFIKFNTTLPSSAPVQQ
LFNSGGNNVNPKGNPLTDEQFEHVLLLRYNSKIISTLE
```

Figure S1. The deduced amino acid sequences of two potential transcripts and one uncharacterized protein LOC569044. The two potential transcripts XM_005168715 and NM_0010838491 were indicated by NCBI (upper and middle panels). The below panel showed the uncharacterized protein LOC569044, which is focused in this study. Yellow color indicated an essential ZBED domain, while green color indicated the XM_005168715 transcript which lacks few amino acids compared with those of NM_001083849.1 and uncharacterized protein LOC569044.

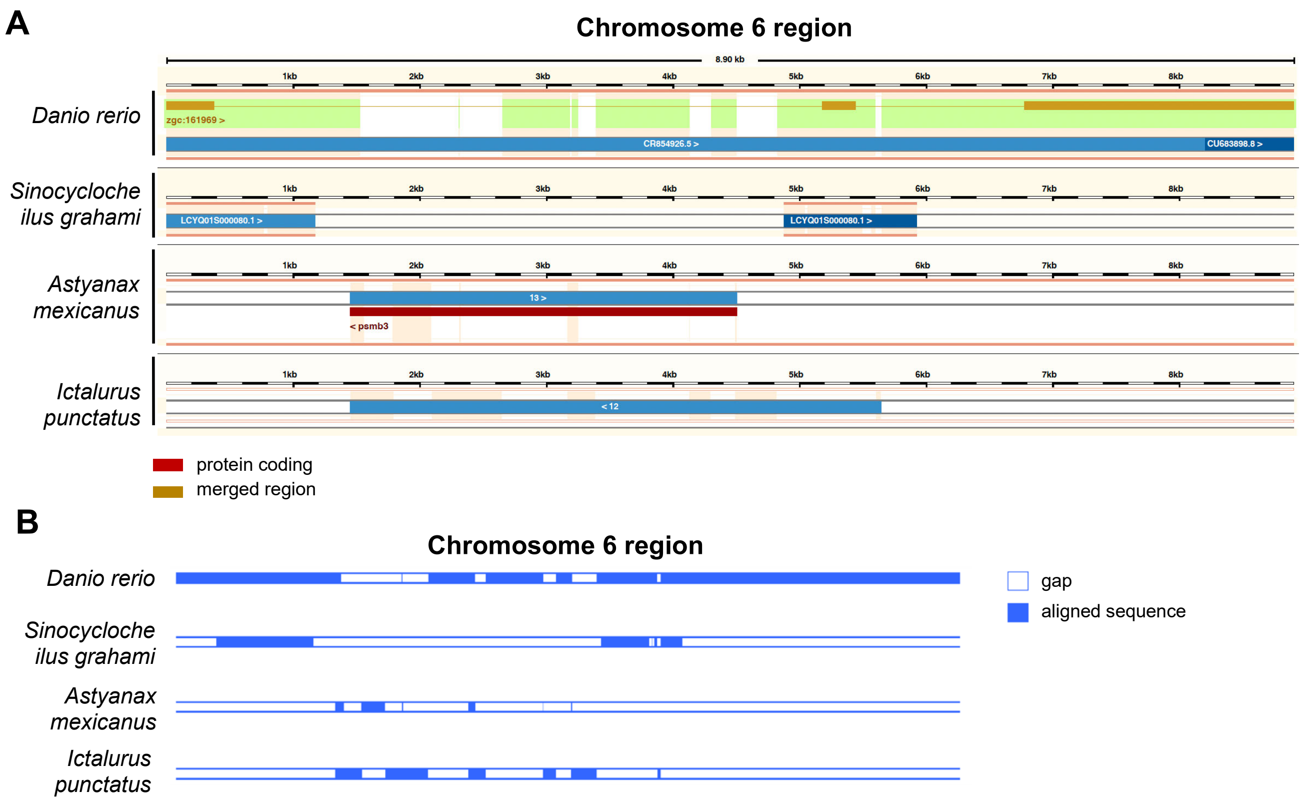

Figure S2. Comparison of the gene maps encoding zebrafish LOC569044 protein and those of multiple bony fishes. (A) *Sinocyclocheilus grahami*, *Astyanax mexicanus* and *Ictalurus punctatus* were found that they were similar alignment sequences located in chromosome 6 region compared to that of zebrafish *zgc:161969*. Horizontal lines indicated chromosome fragments, while dark brown color indicated protein encoded region in the genome. Aligned sequence were indicated in the light brown color and their positions were indicated above in kb. Synteny blocks in fragmented assembly defined by the *Ensembl*. **(B)** The schematic diagram showed the genomic alignments from panel A. The empty blue box indicated non-aligned sequence region, while the blue box indicated aligned sequence region in the genome. This model of synteny conservation is based on the *Ensembl*.
